# Biodistribution of arctigenin-loaded nanoparticles designed for multimodal imaging

**DOI:** 10.1186/s12951-017-0263-8

**Published:** 2017-04-07

**Authors:** Qingxin Cui, Yuanyuan Hou, Yanan Wang, Xu Li, Yang Liu, Xiaoyao Ma, Zengyong Wang, Weiya Wang, Jin Tao, Qian Wang, Min Jiang, Dongyan Chen, Xizeng Feng, Gang Bai

**Affiliations:** 1 College of Pharmacy, State Key Laboratory of Medicinal Chemical Biology, Nankai University, Tianjin, 300071 People’s Republic of China; 2 State Key Laboratory of Medicinal Chemical Biology, College of Life Science, Nankai University, Tianjin, 300071 China; 3 Tianjin Key Laboratory of Tumor Microenvironment and Neurovascular Regulation, Department of Physiology, School of Medicine, Nankai University, Tianjin, 300071 China

**Keywords:** Nanoparticles, Self-assembly, Arctigenin, Localization, Target identification

## Abstract

**Background:**

Tracking targets of natural products is one of the most challenging issues in fields ranging from pharmacognosy to biomedicine. It is widely recognized that the biocompatible nanoparticle (NP) could function as a “key” that opens the target “lock”.

**Results:**

We report a functionalized poly-lysine NP technique that can monitor the target protein of arctigenin (ATG) in vivo non-invasively. The NPs were synthesized, and their morphologies and surface chemical properties were characterized by transmission electron microscopy (TEM), laser particle size analysis and atomic force microscopy (AFM). In addition, we studied the localization of ATG at the level of the cell and the whole animal (zebrafish and mice). We demonstrated that fluorescent NPs could be ideal carriers in the development of a feasible method for target identification. The distributions of the target proteins were found to be consistent with the pharmacological action of ATG at the cellular and whole-organism levels.

**Conclusions:**

The results indicated that functionalized poly-lysine NPs could be valuable in the multimodal imaging of arctigenin.

**Electronic supplementary material:**

The online version of this article (doi:10.1186/s12951-017-0263-8) contains supplementary material, which is available to authorized users.

## Background

Natural products have supplied a myriad of compounds with complex structures as well as diverse biological activities. Conventional natural products research, which involves structural determinations and chemical syntheses of complex natural products, was highly successful until the 1980s. Unfortunately, such research became less productive in the 1990s [1, 2]. It has become significantly more difficult to find the targets of natural products or discover new concepts in chemistry and biology from natural product binding. Therefore, natural products research needs to employ novel strategies based on targets that are involved in biological phenomena and processes [3, 4]. However, it is widely recognized that exploring targets of natural products is an immense undertaking, although many successful methods for identifying targets of small molecules have been reported [5], such as FG-beads technology [6], photoaffinity beads technology [7], the fishing-rod strategy [8], the “compact” molecular probe (CMP) approach [9], the yeast 3-hybrid (Y3H) system [10], and the database-dependent (DBD) strategy [11, 12]. These technologies have conventionally been used for identifying targets of small molecules [13]; however, the most serious failing of these approaches is the difficulty in target tracking in situ [14]. Although the use of quantum dots for tags could solve the problem of target labeling, the fact that these probes are so large that their localization is not totally in situ cannot not be ignored. More importantly, the problems of obtaining the localization information of the target protein from the body, tissue or cells, excluding non-specific protein interference, and elucidating the relationship between the corresponding bioactivity and indications remain a challenge.

Recently, it was discovered that some nanoparticles (NPs) with unique physical properties have wide usage in a variety of fields, such as the life sciences, pharmacology, and the environmental sciences [15–18]. Interestingly, NPs are an important tool, which had drawn significant attention in the fields of molecule imaging and protein capturing [19, 20]. The past 5 years have witnessed several advances that have established luminescent NPs as a highly sensitive technology for real-time optical imaging in small animals [21, 22]. Additionally, recent developments have demonstrated that such NPs could be directed against specific cells through proper surface functionalization with targeting ligands [23, 24]. Despite great effort being devoted to the development of bio-functional self-assembling materials [25], the use of self-assembling NPs in identifying natural product targets is largely unexplored. Indeed, imaging in multiple organisms could prove to be a powerful analytical tool in revealing the targets of natural products.

In this strategy, we used arctigenin (ATG), a phenyl propanoid dibenzyl butyrolactone lignan from *Arctium lappa* L. (commonly known as burdock) as a model compound. It has been widely used in traditional Chinese medicine (TCM) and in Europe and North America for hundreds of years [26, 27]. The biological activities and pharmacological functions reported for ATG include anti-inflammatory, anti-cancer, anti-diabetic, anti-heat shock, antimicrobial and antiviral activities [27–31]. In particular, the target identification process may lead to the discovery biologically intriguing phenomena that could open up new frontiers in chemical biology.

Therefore, we attempted to investigate the targets of ATG using self-assembling NPs. We report here that the use of ATG conjugated poly-lysine fluorescent NPs would be advantageous in such cases; they enable visualization in contrast to affinity beads, and affinity between the ligand and its target is retained. By using the self-assembling NP strategy, we succeeded in imaging the cytosolic target protein of ATG in vivo. This integrated system solved the issue of a natural product interacting with multiple targets and turning on diverse biological activities. This technique may help discover the targets of natural products and benefit drug research and development.

## Methods

### Reagents and materials

Propargylic acid was purchased from Sigma-Aldrich (St. Louis, U.S.A.). N-Hydroxysuccinimide (NHS) and 1-ethyl-3-(3-dimethylaminopropyl) carbodiimide (EDC) were purchased from Alfa Aesar (Massachusetts, U.S.A.). Arctigenin (ATG) was purchased from Phytomarker Co., Ltd. (Tianjin, China). Poly-lysine with an average molecular weight of 20 kDa was purchased from Zhengzhou Bainafo Bioengineering Co., Ltd. (Henan, China). NHS-rhodamine and sulfo-SADP were purchased from Pierce (Rockford, U.S.A.). All other chemicals and solvents used were analytical grade. The water used throughout this work was reagent-grade water produced by the Milli-Q Water Purification System of Nihon Millipore Ltd. (Tokyo, Japan).

### The self-assembly and modification of poly-lysine fluorescent NPs

Briefly, peptide solutions were prepared by dissolving the poly-lysine powder (100 mg, 5 μmol) in 10 mL of phosphate-buffered saline (PBS) buffer (pH 7.4), and then NHS-rhodamine (2 mg, 3.8 μmol) was thoroughly dispersed in an ultrasonic bath. In a reactor equipped with a stirrer, an organic mixture of 200# petrol (13 mL), tetrachloromethane (12 mL), and span-80 (1 mL) (1/1/0.08, v/v/v, respectively) were stirred for 30 min at room temperature. The mixture of the organic and peptide solutions was thoroughly dispersed using a homogenizer (FA25, Fluco, England), added to the reactor, and dispersed at 12,000 rpm. Subsequently, petroleum ether was added slowly to the mixture. Immediately after the delamination process, PBS solution was added, and self-assembly occurred during the biphasic equilibrium process to room temperature. The aqueous phase was collected, and the process was repeated 3 times to obtain poly-lysine fluorescent NPs. The crude product was centrifuged for 10 min at 1000 rpm, and the precipitate was discarded. Finally, the supernatant was centrifuged for 10 min at 12,000 rpm. The supernatant fluid containing the poly-lysine fluorescent self-assembling NPs was stored at 4 °C.

**Fig. 1:**
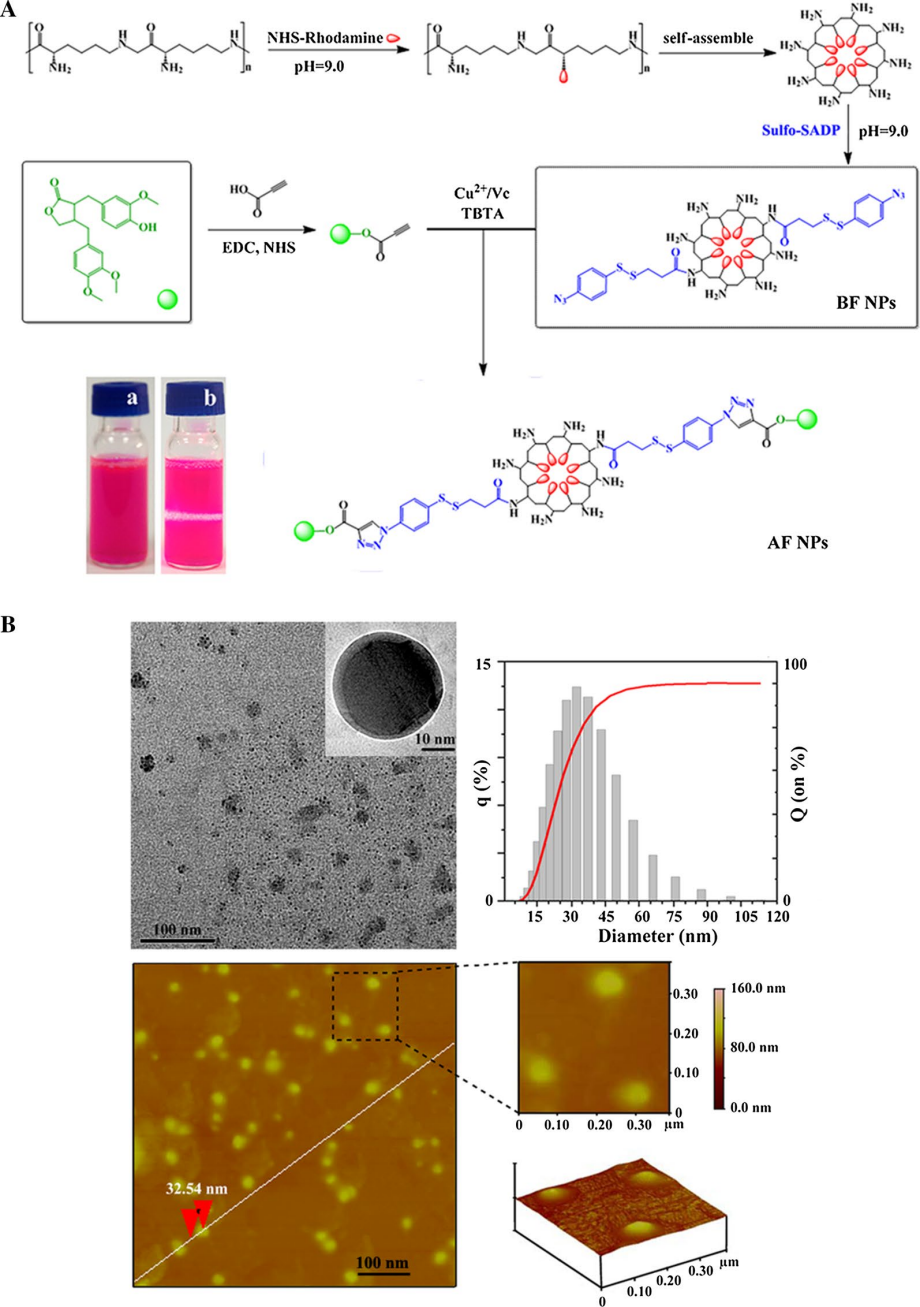
The characterization of NPs. **A** The general strategy for the synthesis of blank fluorescent NPs (BF NPs) and ATG conjugated poly-lysine fluorescent NPs (AF NPs). **B** TEM images of AF NPs; *scale bar* 100 nm. The particle size distribution of AF NPs was determined; the mean diameter of the NPs was 33 nm. AFM images of AF NPs were obtained; the height of the NPs was 32.54 nm

Sulfo-SADP (15 mg, 0.03 mmol) was dissolved in 0.2 mL of borate buffer (0.2 mol/L pH 8.0), and 1 mL poly-lysine fluorescent NPs was added. The mixture was stirred at room temperature for 4 h. The azide functionalized poly-lysine fluorescent NPs were collected by ultrafiltration (Millipore, USA) and washed with 20 mL of phosphate-buffered saline (PBS, pH 7.4) several times until sulfo-SADP could no longer be detected in the filtrate. The general strategy for synthesis of blank fluorescent NPs (BF NPs) is shown in Fig. 1A.

### Drug conjugation to poly-lysine fluorescent NPs

The synthesis of alkynyl-ATG is depicted in the electronic Additional file 1 (scheme 1). Alkynyl-ATG (17 mg, 0.04 mmol) was dissolved in 0.2 mL of borate buffer (0.2 mol/L pH 8.0), and 2 mL of azide BF NPs was added, followed by the catalyst (0.2 mmol/L Tris-triazoleamine, 1.0 mmol/L CuSO_4_, and 2.0 mmol/L sodium ascorbate). The mixture was stirred at room temperature for 1 h. The functionalized NPs were was added to a 50 mL 3kD ultrafiltration tube and washed with 20 mL of PBS several times until small molecules could no longer be detected in the filtrate. The general strategy for synthesis of ATG bearing poly-lysine fluorescent NPs (AF NPs) is shown in Fig. 1A.

### Characterization of NPs

A 10-μL portion of the sample solution was placed on newly clipped mica and washed with 1 mL of distilled water and then air-dried. The samples were examined using an atomic force microscope (AFM, Veeco Company, Multimode, NanoIIIa) in tapping mode in air at room temperature. The morphology and structure of the functionalized NPs were studied using a transmission electron microscope (JEOL JEM-2010, USA). The particle hydrodynamic size was measured using a Counter laser size analyzer (Beckman Coulter, USA). The PEI coating of the NPs was checked using a Perkin-Elmer (Norwalk, USA) Spectrum GX Fourier transform infrared (FT-IR) spectrometer (Nicolet NEXUS 670) and KBr pellets. The chemical and physical stability of AF NPs was evaluated during 1 month storage at 4 °C, in terms of mean particle size, polydispersity index(PDI) and zeta potential. Visual inspection was also employed to detect any crystallization process or mold formation.

### Zebrafish imaging in vivo

Zebrafish (AB strain) were cultured under a 14 h light/10 h dark cycle at 28.5 °C. Fertilized eggs were collected and cultured in standard system water (KCl 0.05 g/L, NaHCO_3_ 0.025 g/L, NaCl 3.5 g/L, and CaCl_2_ 0.1 g/L, with 1 mg/L methylene blue, pH 7.0) until 4 days post fertilization (dpf). Zebrafish larvae at 4 dpf were exposed to BF NPs and AF NPs for 1, 2 and 3 h, respectively. Zebrafish were anesthetized by immersing in tricaine for bioimaging. The specimens were photographed with a fluorescence stereomicroscope (OLYMPUS SZX 10, Japan) fitted with a digital camera. The parameters of the camera remained unchanged when taking pictures for the untreated control groups, BF NP groups and AF NP groups. The larvae of each group were collected and fixed in 4% paraformaldehyde (pH 7.0) for tissue sectioning.

### Cell culture and animals

The BEAS-2B cell line, derived from human bronchial epithelial cells, was obtained from the American Type Culture Collection (Rockville, MD). BEAS-2B cells were cultured using DMEM/F12 medium that was supplemented with 10% FBS, 100 U/mL penicillin and 0.1 mg/mL streptomycin and maintained at 37 °C in 5% CO_2_. The medium was replenished every 3 days, and the cells were sub-cultured once they reached 80–90% confluence. Male Kunming mice (20–22 g) were purchased from the Beijing Vital River Laboratory Animal Technology Co., Ltd. (Beijing, China). All animals were kept in an animal room with a temperature of 23 ± 2 °C, a humidity of 60 ± 5%, and a 12-h dark to light cycle, with free access to food and water. The animals were housed under the above conditions for a 3-day acclimation period.

### Animal model of acute lung injury (ALI) and treatment

Thirty animals were randomly divided into three groups—blank, control and model groups—each containing ten mice. Lipopolysaccharide (LPS) (5 mg/kg) was administered intratracheally to induce ALI. NPs (10 mg/kg) were intravenously injected 1 day after LPS administration [29]. The chosen doses of these drugs were based on our previous studies and preliminary experiments.

### Mouse imaging in vivo

The mice were anaesthetized intraperitoneally with chloral hydrate (10 g/kg). Chronic bronchitis was induced by endotracheal instillation of LPS, and this was followed by NP injection (0.2 mL) through the caudal vein 1 day later [32]. After the disease was successfully induced, the animals were randomly divided into two groups. Model groups received AF NPs, intravenous injection (i.v.), and were subjected to in vivo imaging continuously for 1 h. Control groups received BF NPs, and the above procedure was repeated. The excitation wavelength was 550 nm, and the emission wavelength range was 600–700 nm.

The mice (n = 10) were sacrificed at pre-determined time points immediately after the imaging procedure. Blood was dropt by heart puncture. Then a peristaltic pump with saline flushing was used to wash the vessels through the ventricles until the eluate was colorless. The organs (heart, liver, lung, kidney, and spleen) of the control and model groups were harvested and cooled in liquid nitrogen. Then, the organs were ground repeatedly until they were powdered. Anhydrous ethanol was added to the powder, and it was sonicated for 5 min in ice-cold water. Then, the mixture was centrifuged for 10 min at 12,000 rpm. The supernatant fluid containing rhodamine-bound poly-lysine polymers was collected, and these were detected using a luminoscope (Tecan Safire 2 type, Switzerland). The excitation wavelength was 550 nm and the emission wavelength 592 nm. A part of the lung tissue was harvested and quickly fixed in 4% paraformaldehyde. The lung tissue was dehydrated, paraffin embedded, and sliced. After hematoxylin and eosin (H&E) staining of lung tissue slices, pathological changes were observed using a CKX41 microscope (Olympus Co., Japan).

### Cellular localization of poly-lysine fluorescent NPs

BEAS-2B cells were washed with pre-cooled PBS and treated with 100 μL of AF NPs or BF NPs. After 3 h of incubation, the cells were washed with PBS, fixed using 4% paraformaldehyde, and permeabilized with 1% Triton X-100 for 30 min. Then, the cells were labeled with DAPI, and fluorescence images were obtained with a confocal microscope (TCS SP5, Leica, Germany). The excitation wavelengths were 365 and 552 nm, and the emission wavelengths were 465 and 590 nm, respectively.

In parallel, cells were incubated at 37 °C for 6 h, and then, 0.25% trypsin was added (Gibco, 25200-056, final concentration 0.05%), and the sample was incubated for 1 min. The cell suspension was washed in DMEM/10% FBS to inactivate trypsin, followed by two washes in PBS. Digested cells were then resuspended in Hank’s balanced salt solution containing 1% bovine serum albumin, 2 mM EDTA (FC buffer) and kept at 4 °C before flow cytometry analysis.

Two aliquots were placed in tubes for flow cytometry analysis. Fluorescence-minus-one controls were generated for the NPs used and compensations were set using BD Plus CompBeads and FACSDiva software. Data collection was performed using a Becton–Dickinson LSR II flow cytometer with ultraviolet (20 mW), violet (25 mW), blue (20 mW) and red (17 mW) lasers with the default filter configuration and utilizing the High Throughput Sampler attachment. At least 10,000 events were collected per well. The results were analyzed using Cell Quest software (Becton–Dickinson Co., USA). FCS 3.0 files were exported to Flow Jo version 9.3. In all cases, dead cells and doublets were excluded prior to analyzing marker expression.

## Results

### NP molecular design and morphology assay

Self-assembling NPs have been extensively explored as an efficient carrier for fluorescent probes and drug delivery due to their potential ability to label the target. We therefore designed and synthesized azido functionalized poly-lysine fluorescent NPs and alkynyl modified ATG for drug loading. The proposed ATG carrying fluorescent NPs (AF NPs) were formed based on click chemistry. The poly-lysine in the NPs might have two functions. One is to increase the hydrophilicity of the resulting peptides for self-assembly. The other is to endow the self-assembling NPs with active amino-groups to link fluorescence dyes and functional groups. The alkynyl modified ATG could be easily obtained by standard organic synthesis and was purified by HPLC. The purity and identity of the compound was determined by ^1^H NMR and ^13^C NMR (see Additional file 1: Figure S1).

After successful synthesis, the morphology of the self-assembling NPs was characterized by TEM. As shown in Fig. 1B, poly-lysine self-assembled into uniform NPs and showed a regular spherical structure. Figure 1B presents the particle size distribution of the NPs; the mean diameter was approximately 33 nm, which was consistent with the results of TEM analysis. The AFM images of the AF NPs are shown in Fig. 1B. The similar morphologies observed in the AFM images are noteworthy. The height of each NP, which as approximately 32.54 nm, was similar to that observed with TEM. These results suggested that functionalization of the poly-lysine NPs had negligible effects on their self-assembling properties and morphologies. The results of stability studies of AF NPs during 1 month storage at 4 °C were stable, maintaining nanometric dimensions of the dispersed particles and PDI and Zeta potential values similar to those of the corresponding freshly prepared systems. It suggested the good physical–chemical stability and highlighting the preservative effect of poly-lysine.

### Zebrafish imaging in vivo

**Fig. 2:**
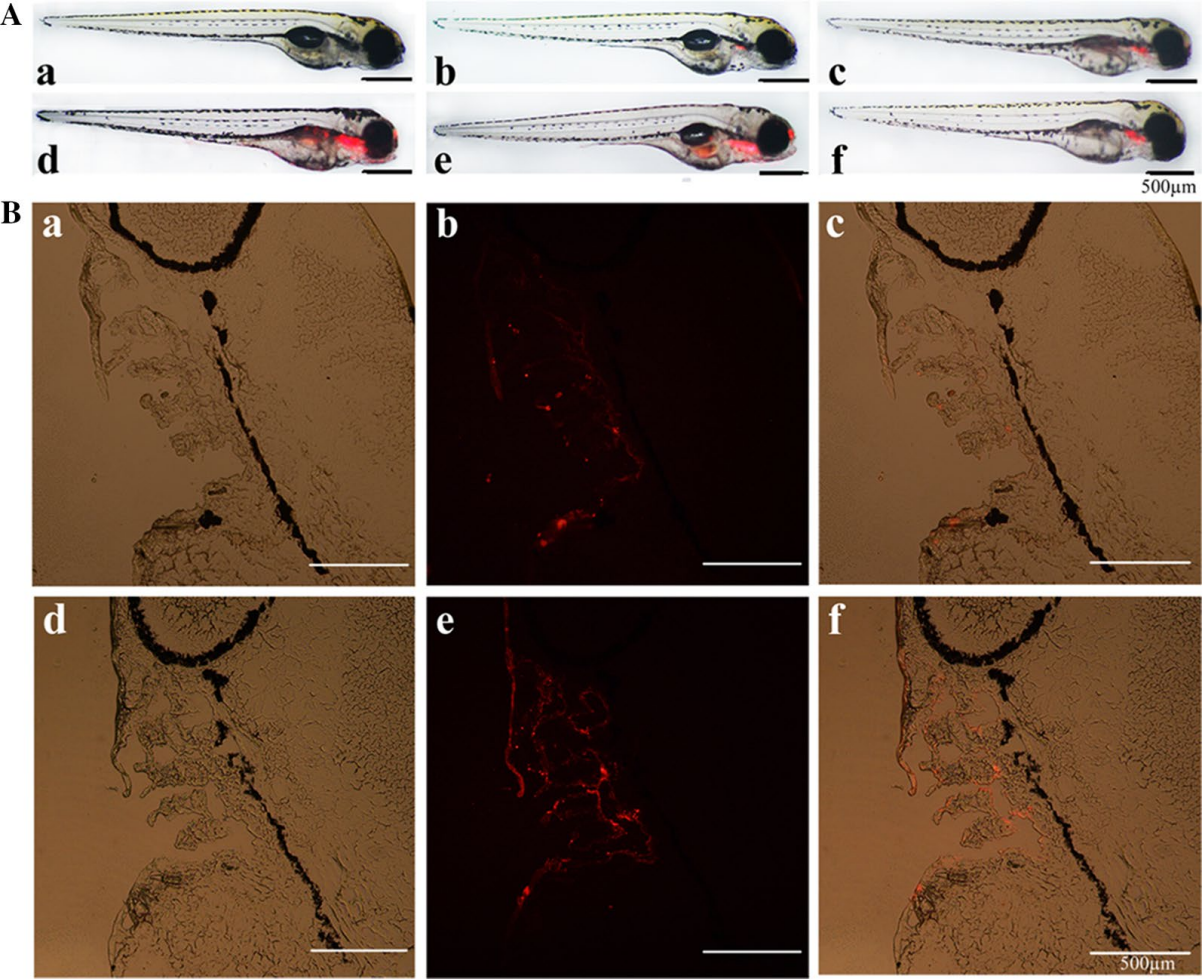
BF NP and AF NP bioimaging in 4 dpf zebrafish in vivo. **A** The untreated control group is represented in *a*; *b* and *c* represent BF NPs treated for 1 and 2 h, respectively; *d*, *e* and *f* represent AF NPs treated for 1, 2 and 3 h, respectively. **B** Sections from the treated groups of BF NPs (*a*–*c*) and the treated groups of AF NPs (*d*–*f*) at 2 h post treatment are shown. The *scale bar* indicates 500 µm

To examine the tissue localization of NPs in zebrafish, both BF NPs and AF NPs were added to standard system water for 1–3 h, and they were visualized in vivo by fluorescence detection. It was apparent that in the non-NP-treated control group, development was not disturbed, and no fluorescence was detected, whereas other groups of zebrafish showed specific fluorescence (Fig. 2A). Interestingly, the fluorescence intensity of BF NP-treated zebrafish remained almost unchanged from 1 to 2 h. Notably, the fluorescence intensity was significantly clustered and increased with an increase in AF NP incubation time. Tissue localization analysis showed that the fluorescent AF NPs were present as an intense red on the gills of the zebrafish, especially in the epithelium of the branchial arch, as shown in Fig. 2B. In contrast, with BF NPs, a weak fluorescence was observed 2 h after treatment. These results clearly indicated that AF NPs were ideal carriers in developing a suitable method for target identification, as they were specifically located on the gills of the zebrafish.

### Evaluation of targeting specificity in vivo

**Fig. 3:**
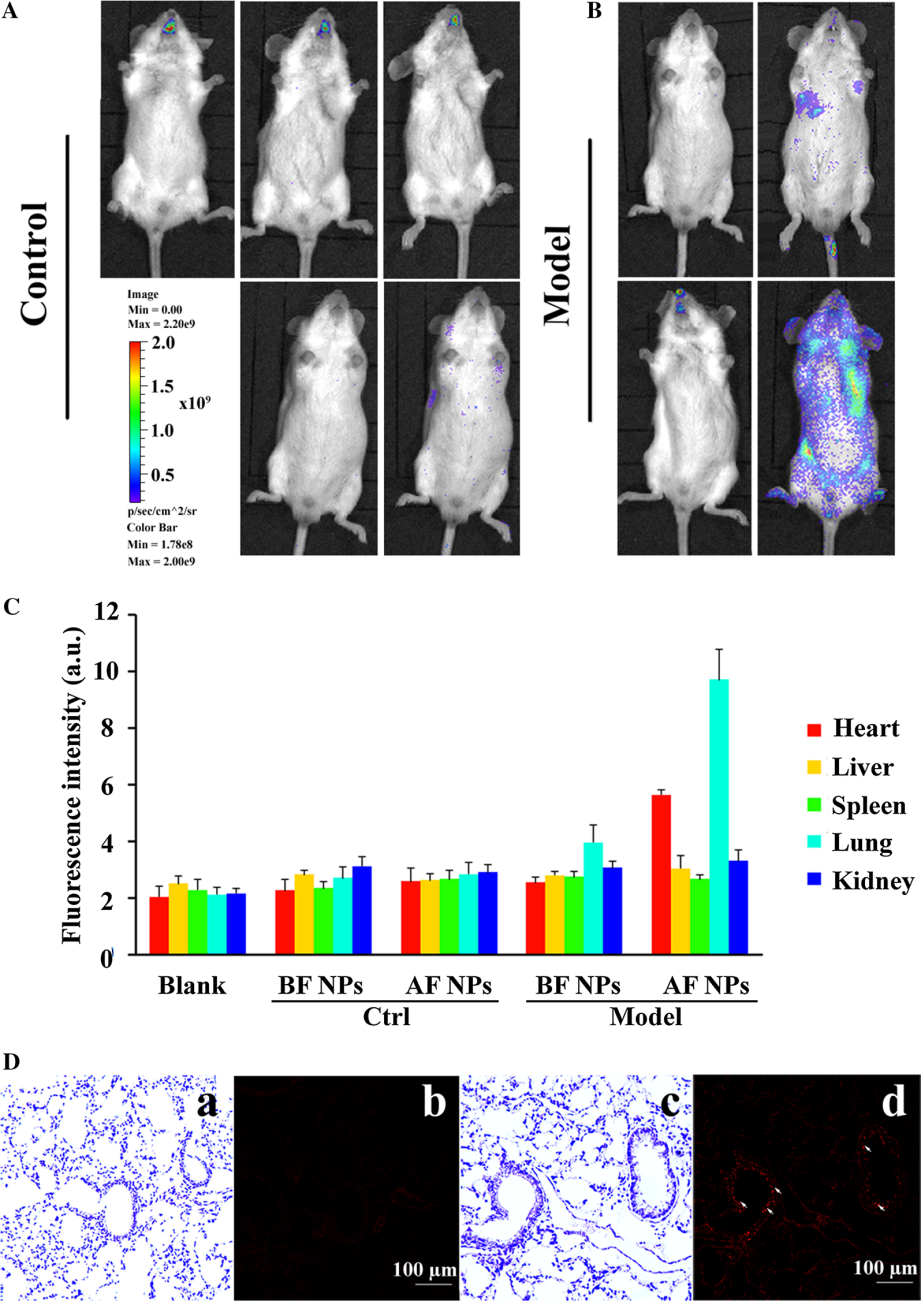
BF NP and AF NP bioimaging in mice in vivo. **A** Representative images from control mice. The *bar plot* shows the distribution of fluorescence signal intensity. **B** Representative images from model mice. **C** *Bar plots* represent the concentrations of NPs in different tissues. The values shown represent the mean values ± sem. **D** Immunohistochemical staining was performed using diverse types of lung tissue

The targeting specificity of AF NPs was determined by fluorescent in vivo imaging. BF NPs and AF NPs were administered to control and LPS-induced pneumonia model mice, respectively, via tail vein injection. After 30 min of observation, only AF NPs were found enriched in the model group (Fig. 3B), and especially in the thoracic cavity. There was also a slight enrichment of BF NPs at the same site. No significant differences were found in the organs of the control group (Fig. 3A). To further evaluate their tissue distribution, NPs were isolated from the organs. The fluorescence intensity of rhodamine from the different organs was determined and is presented in Fig. 3C. There were significant differences in fluorescence intensity between the groups. Compared with the control group, LPS stimulation resulted in a prominent increase in the lung (P < 0.01) and a slight increase in the heart sizes (P < 0.05). However, other organs showed no significant changes (P > 0.05). Additionally, the liver, spleen and kidneys showed no significant changes in morphology after the injection of AF NPs. H&E staining of lung slices (Fig. 3D) indicated greater numbers of AF NPs in the bronchial epithelial cells of the lung in the model group (indicated as arrows in Fig. 3D). BF NPs were not found in lung slices in the model group indicating that the specific targets of AF NPs were inflammation-associated epithelial cells in the lung tissue.

### Cross-membrane uptake of AF NPs

**Fig. 4:**
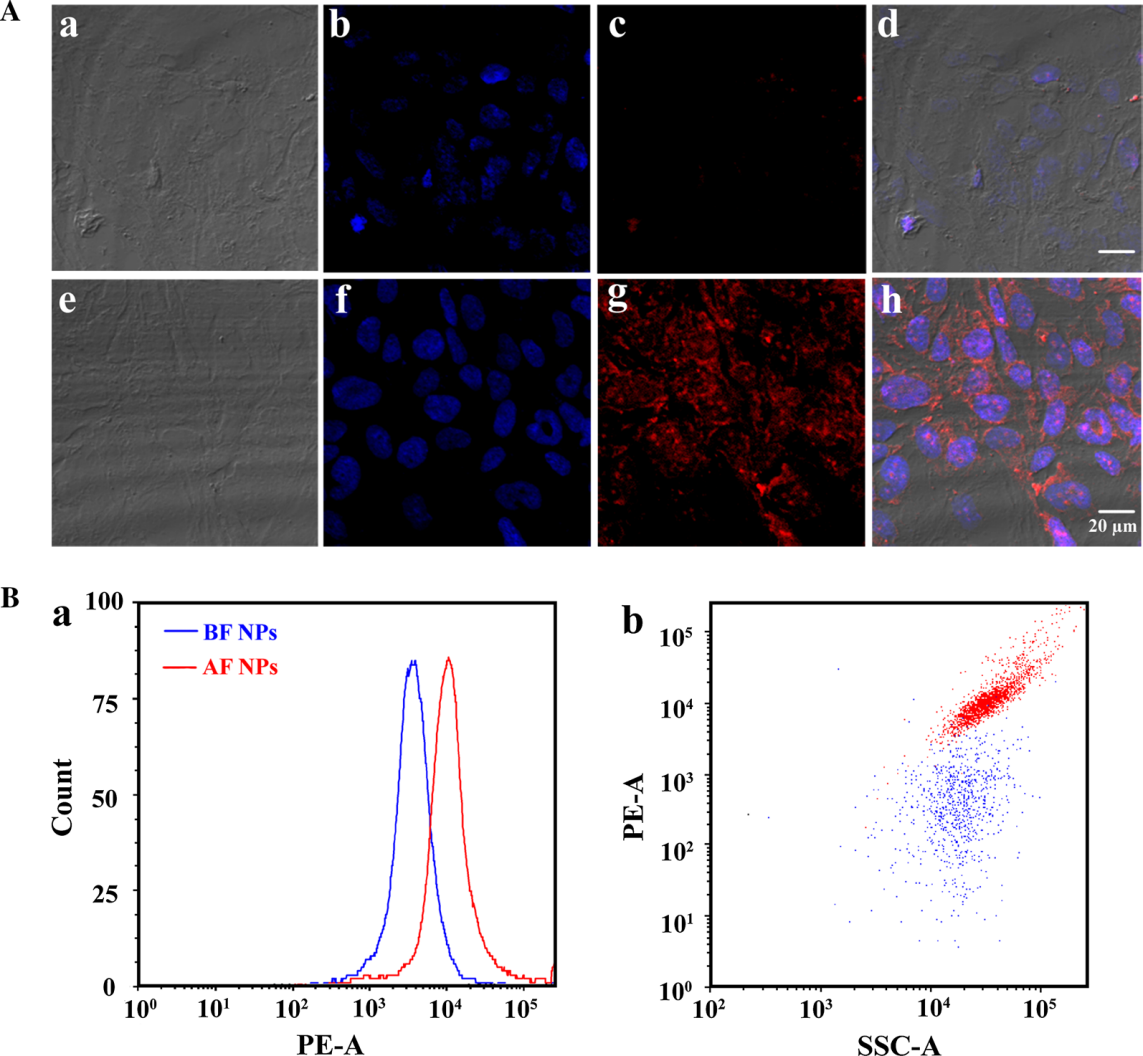
Target localization using BF NPs and AF NPs. **A** General strategy for fluorescent labeling of the target protein. BEAS-2B cells were treated with AF or BF NPs, followed by the DAPI fluorescent marker. Fluorescence and DIC images are shown. All *scale bars* 20 μm. **B** The histogram of flow cytometry analysis is shown in *a*. The scatter diagram of flow cytometry analysis is shown in *b*

We investigated the ability of AF NPs to cross the membrane barrier and bind to its targets in vitro (Figs. 4A a, e). For this purpose, DAPI (4′,6-diamidino-2-phenylindole) and then NPs were added to the culture medium of BEAS cells, which were imaged by confocal microscopy (Figs. 4A b, f). Treatment with BF NPs did not result in effective internalization in cultured BEAS-2B cell lines (Fig. 4A g). However, the fluorescence signal could be clearly visualized in AF NP-treated BEAS-2B cells, indicating their internalization by the cells (Fig. 4A c). As shown in Figs. 4A d, h, our data demonstrated the ability of AF NPs to cross the membrane and bind with the target protein in the cytoplasm. The results of flow cytometry analysis suggested that the fluorescence intensity of rhodamine positive cells was markedly increased from 2 × 10^3^ in the BF NP group to 10^4^ in the AF NP group. Interestingly, the side scatter (SSC) of both groups remained almost unchanged (Fig. 4B). These analyses showed that the AF NPs were highly specific and could be a reliable tool for targeted protein capture.

## Discussion

As is generally known, tissue targeting can be achieved by taking advantage of the enhanced permeability and retention time resulting from a chaotic tissue microenvironment [33]. The molecules in circulation cannot reach the tubule until they are filtered by the glomerular filtration barrier. It is estimated that the mean diameter of the glomerular filtration barrier is approximately 40–60 nm. The sizes of the target NPs should not exceed this range [34]. Fortunately, multiple observations confirmed that the mean diameter of our AF NPs was approximately 33 nm, and grafting with poly-lysine preserved its biocompatibility. Furthermore, the concept is based on ATG itself having high affinity for its target and modification of a functional probe to keep its original affinity intact. Structure–activity relationship studies have shown that modifications at the hydroxyl group of ATG had a limited impact on its biological activity [35]. Therefore, the combination of alkynylated ATG and azide-modified NPs with introduction of rhodamine by copper-catalyzed azide-alkyne cycloaddition (CuAAC) [36] was expected to give a good signal-to-noise ratio in target identification experiments. The present approach is different from traditional magnetic protein capture, enrichment or fluorescent NP location tracking [37] and provided optimum results in which not only the specific target was traced but also enriched by the same AF NPs tool. The experiments demonstrated that ATG-bound self-assembling fluorescent NPs are a highly sensitive optical tool to monitor the target in real time and are especially useful for intracellular target protein enrichment [38, 39].

Fluorescence tracking showed that ATG conjugation effectively eliminated non-specific distribution and improved the tissue-targeting efficiency. Interestingly, the AP NPs localized in the epithelium of the branchial arch in zebrafish. This result is somewhat at odds with the observation that the lung is the major site of ATG localization under standard physiological conditions [29].We also noticed a seemingly selective biodistribution in the lung of the LPS-induced pneumonia mouse. This result is in good agreement with a large body of pharmaceutical research on ATG; the fruits of *Arctium lappa* L. are an often-used herbal drug in TCM for the treatment of respiratory diseases [31]. Lung homing of ATG, as determined by H&E staining and analysis of tissues, is most certainly associated with a rapid stress reaction in the lung capillary bed and is typical of tissue targeting based on enhanced permeability.

An important problem observed in target identification using molecular probes is that the presence of linker and tag moieties may decrease the affinity with the target as well as cause nonspecific adsorption. Reduction of nonspecific binding to proteins is important for the success of target identification approaches.

The mechanism of action of ATG appears to be multifaceted but was not fully understood previously. It has been proposed that the immunomodulatory effects of ATG were partly responsible for its anti-inflammatory activity [40–42] as well as its therapeutic value in the treatment of acute lung injury [29, 31]. Identification and validation of targets for bioactive small molecules is important in chemical biology and drug development. Recent technological developments and innovations have contributed significantly to these efforts. Herein, we focused on an approach involving a novel herbal remedy conjugated to poly-lysine fluorescent self-assembling NPs that enables the tracking of bioactive compounds to detect cytosolic targets.

## Conclusions

The strategy was shown to be effective for the synchronous tracking of targets. Additionally, our results indicate that the identified targets of ATG are strongly linked with its pharmacologic action. Although the generality of this approach needs to be verified by testing many other molecules, the results suggest the utility of the NP approach in increasing the success rates of the biochemical labeling of protein targets of natural products. It is important to choose other appropriate methods depending on the small molecule; multiple approaches may be necessary to accelerate target identification and understand the mode of action of investigative compounds.

## Additional file

**Additional file 1.** Supplementary information about alkyne-ATG synthesis, purification, characteristics and FI-IR analysis of functionalized NPs.
