# Supplementary material for: Biodistribution of arctigenin-loaded nanoparticles designed for multimodal imaging
Source: J Nanobiotechnology. 2017 Apr 7;15:27. doi: 10.1186/s12951-017-0263-8 (PMC5383946; doi:10.1186/s12951-017-0263-8)
Supplement: Supplementary file 1 — Additional file 1. Supplementary information about alkyne-ATG synthesis, purification, characteristics and FI-IR analysis of functionalized NPs. [file 12951_2017_263_MOESM1_ESM.docx]

**Supporting information**

**Scheme 1: Synthesis of Alkynyl-arctigenin (Alkynyl-ATG)**

Figure S1 The synthetic strategy of arctigenin-ATG

To a stirred solution of arctigenin(ATG) (compound 1, 0.372g, 1 NPol, 1 equiv) and 4-dimethylaminopyridine (DMAP, 0.013g, 0.1 NPol) in CH_2_Cl_2_, 20 mL of EDC (0.385 g, 2 NPol) was added, and the mixture stirred at room temperature for 12 hours. The reaction mixture was diluted with CH_2_Cl_2_ and washed with 1 N aq HCl, 5% aq NaHCO_3_, and then brine. The organic layer was gathered and dried by anhydrous Na_2_SO_4_, filtered, and concentrated under reduced pressure to give the Alkynyl-ATG (compound 2, 0.262 g, 64%) as white solid. Rf =0.7 (PE / EtOAc 1:1); ^1^H NMR [CDCl_3_, 400 MHz] 6.98-6.94(m, 1H), 6.79-6.75(m, 2H), 6.68-6.65(m, 1H), 6.58-6.50(m, 2H), 3.84(s, 3H), 3.81(s, 3H), 3.76-3.74(m, 2H), 2.97-2.95(m, 2H), 2.83(s, 1H), 2.66-2.48(m, 4H). ^13^C NMR [CDCl_3_, 100 MHz] δ 35.3, 38.6, 41.2, 46.7, 56.5, 56.5, 56.6, 72.4, 73.5, 76.7, 111.6, 112.6, 112.7, 121.1, 122.5, 122.6, 131.9, 135.6, 136.5, 140.5, 147.1, 149.5, 151.2, 178.0.

**Characterization of optimally functionalized alkynyl NPs**

Figure S2 FT-IR analysis of each step of functionalized NPs

NPs were synthesized step-by-step according to the scheme described in the article. The PEI coating of the NPs was checked using a Perkin-Elmer (Norwalk, USA) Spectrum GX Fourier transformation infrared (FT-IR) spectrometer (Nicolet NEXUS 670) using KBr pellets. FT-IR spectroscopy was used to confirm the functionalized NPs by characterizing the properties of the functional groups on the NP surfaces. In Figure 2, BF NPs, the -CONH bond at 1664 cm^-1^ and 1540 cm^-1^ also verified the presence of polylysine on the surface of NPs, 3536 cm^-1^ indicated the existence of free amino groups (Figure 2A, BF NPs). Successful attachment of Rhodamine was confirmed by the lack of free amino groups at 3536 cm^-1^. (Figure 2B, Rhodamine NPs). Finally, the existence of functionalized azide groups was confirmed by the small peak at 2086 cm^-1^. The results from FT-IR spectroscopy showed that azide functional groups on NP surfaces were synthesized successfully.
